# 610 Inclusion of People Experiencing Homeless in Research Programs: Strategies Derived from Community Participatory Action Research

**DOI:** 10.1093/jbcr/iraf019.239

**Published:** 2025-04-01

**Authors:** Deja Nicholas, Geun-woo Oh, Caitlin Orton, Carly Marincasiu, Maiya Pacleb, Leslie Enzian, Barclay Stewart

**Affiliations:** Harborview Medical Center; Harborview Medical Center, University of Washington; University of Washington; Harborview Medical Center, University of Washington; UW Medicine Regional Burn Center; Harborview Medical Center, University of Washington; University of Washington

## Abstract

**Introduction:**

People experiencing homelessness (PEH) face a higher risk of burn and cold injuries compared to the general population. Additionally, PEH experience worse health and function after injury due to intersectional socio-health disparities. Research programs generally exclude PEH or are not designed to be inclusive of their unique needs that promote participation. Therefore, we aimed to understand how research programs might be more inclusive of PEH to ensure that study findings and interventions can be generalized to this important stakeholder group.

**Methods:**

We interviewed 12 PEH living with burn or cold injuries. We leveraged the Trauma-Informed, Socially Just Research Framework and Levesque Framework for healthcare access to develop an interview guide that aimed to better understand how PEH prefer to engage with research programs. Interview recordings were transcribed, and two coders used inductive and deductive approaches for analysis using grounded theory.

**Results:**

Participants reported a range of strategies to enhance research participation and retention. We identified 5 key themes: (1) Ensure clarity and acknowledge literacy concerns when communicating – improve understanding of roles, expectations and timelines; (2) Focus on research questions that closely reflect the priorities of PEH – integrate PEH into research design rather than including them only as an ‘observed’ group; (3) Use trauma-informed care during approach and follow-up encounters – build relationships with PEH to maximize enrollment and retention; (4) Use multiple methods/media when communicating with PEH – mitigate issues related to unreliable access to phones, data plans, internet and transportation; (5) Incentivize time and effort – offer population-specific incentives (e.g., phone data, public transportation cards, money) and have a variety to choose from demonstrates attention to issues that matter among PEH.

**Conclusions:**

These findings emphasize the need for creating specific research protocols and pathways with people experiencing homelessness (PEH) that prioritize clarity, meaningful participant engagement, relationship-building, and respect. Additionally, incorporating a trauma-informed and harm reduction approach is essential to optimally engage this study population. Addressing communication and transportation challenges and offering flexible, personalized incentives may further enhance participation and retention among PEH.

**Applicability of Research to Practice:**

These strategies will ensure inclusion of PEH in research programs. By doing so, study findings and interventions may be more generalizable to PEH.

**Funding for the Study:**

The contents of this abstract were developed under a grant from the National Institute on Disability, Independent Living, and Rehabilitation Research (NIDILRR grant #90DPBU0005). NIDILRR is a center within the Administration for Community Living (ACL), Department of Health and Human Services (HHS). The contents of this abstract do not necessarily represent the policies of NIDILRR, ACL, HHS, and do not assume endorsement by the Federal Government.

